# Tartaric Acid Exacerbates DSS-Induced Colitis by Promoting Eosinophilic Inflammation via IL-13 and IL-5Rα Upregulation

**DOI:** 10.3390/pathogens14040366

**Published:** 2025-04-08

**Authors:** Bushra Riaz, Hye-Myung Ryu, Bunsoon Choi, Seonghyang Sohn

**Affiliations:** 1 Department of Biomedical Sciences, Ajou University School of Medicine, Suwon 16499, Republic of Korea; bushra.riaz148@gmail.com; 2 Department of Microbiology, Ajou University School of Medicine, Suwon 16499, Republic of Korea; naya101@hanmail.net; 3 Institute of Medical Science, Ajou University School of Medicine, Suwon 16499, Republic of Korea; blueppang@aumc.ac.kr

**Keywords:** eosinophils, Th2 cytokines, tartaric acid, colitis, mouse model

## Abstract

Eosinophils are granulocytes involved in the effector phase of type 2 T cell immune responses, which are elevated in inflammatory conditions like ulcerative colitis (UC) and other allergic diseases. UC is a chronic inflammatory colon disease, marked by excessive eosinophil infiltration and elevated Th2 cytokines, which contribute to mucosal inflammation and tissue damage. Dietary factors, including certain organic acids, can influence UC progression by modulating gut immune responses. This research is the first to explore the dose-dependent effects of tartaric acid (TA), a naturally occurring organic acid widely used in the food industry, on eosinophil activation and Th2 cytokine response in both normal mice and a dextran sulfate sodium (DSS)-induced colitis model. Normal mice were treated with TA at varying doses (5 µg, 25 µg, and 50 µg/mouse/day), while colitis mice received 50 µg TA. Eosinophil activation markers (CD11b+, SiglecF+, and CCR3+), Th2 cytokines (IL-4, IL-13, and IL-31), and IL-17 were assessed in peripheral blood leukocytes, lymph nodes, and splenocytes using flow cytometry. Additionally, mRNA expression levels of eosinophil-associated chemokines and cytokines in the splenocytes were quantified with real-time qPCR. Our results demonstrate a dose-dependent effect of TA, with the highest dose (50 µg) significantly increasing eosinophil activation markers, Th2 cytokines, IL-17, and mRNA expression of SiglecF, CCL11, and toll-like receptor 4 in normal mice. In colitis mice, treatment with 50 µg TA showed marked increases in IL-13 levels compared to those of untreated colitis mice, reflecting increased eosinophil recruitment to inflamed tissues. Moreover, mRNA expression of IL-5Rα was elevated in normal mice and colitis mice administered with TA. These results suggest that TA enhances eosinophil proliferation, the upregulation of their regulatory molecules, and Th2 immune profiles, potentially worsening the severity of colitis.

## 1. Introduction

Eosinophils are myeloid-derived innate immune cells essential for type 2 T cell (Th2) responses [1]. They primarily reside in mucosal tissues, blood, lymph nodes (LNs), the spleen, and bone marrow and contribute to various homeostatic functions [2]. Eosinophils, characterized by a bilobed nucleus and granules containing cytokines and enzymes, express surface markers such as CD11b, SiglecF, CC-chemokine receptor 3 (CCR3), and the interleukin (IL)-5 receptor alpha (IL-5Rα) [3,4,5]. They are implicated in various inflammatory and allergic diseases, including ulcerative colitis (UC), asthma, atopic dermatitis (AD), and eosinophilic esophagitis [6,7,8,9]. UC is a chronic inflammatory bowel disease (IBD) affecting the colonic mucosa, characterized by persistent superficial inflammation confined to the colon [10]. Elevated eosinophil counts in patients with UC are associated with disease severity, and dietary components such as organic acids may contribute to this progression by modulating the gut immune responses [11,12,13]. Understanding eosinophil accumulation is essential for elucidating the pathogenesis of eosinophil-related disorders and developing targeted treatments.

Alpha hydroxy acids (AHAs), such as citric acid, tartaric acid (TA), and lactic acid, are widely used in cosmetics. At high concentrations, AHAs can be irritating, whereas at low concentrations, they may provide benefits through epigenetic modifications in the inflammasome complex [14]. TA is a naturally occurring organic acid found primarily in fruits and beverages such as wine and has recently attracted attention for its potential immunomodulatory properties. TA is best known for its antioxidant and antimicrobial activities when consumed in adequate amounts [15]. However, at higher concentrations, TA may exhibit toxic effects [16]. Emerging evidence suggests that TA may influence immune responses, although its role in immune regulation and disease exacerbation is still unclear and requires further study.

Th2 cells mediate eosinophilic inflammation by secreting IL-4, IL-5, IL-13, and IL-31, which promote eosinophil development, survival, activation, and recruitment into inflamed tissues, thereby exacerbating inflammation [17,18,19]. In the inflamed intestine, eosinophil migration and activation are promoted by the Th2-mediated immune responses [20]. Inappropriate Th2 and Th17-mediated immune responses have been detected in UC patients. Th2 cells producing IL-13 and Th17 cells producing IL-17 are commonly present in the lamina propria mucosae, contributing to UC pathology [21]. However, whether TA is involved in the modulation of these cytokines remains unknown. Understanding how these cytokines are modulated in response to TA treatment in both normal and colitis mice may help elucidate the mechanisms underlying colitis, allergic reactions, and other eosinophil-related diseases.

Eosinophils possess receptors for chemokines, adhesion molecules, and cytokines, which regulate homeostasis, inflammation, and immune responses [17,22]. CCR3, a key receptor on eosinophils and a subset of Th2 lymphocytes [23,24,25], facilitating eosinophil recruitment. CCR3 knockout mice have been shown to exhibit reduced eosinophil infiltration and Th2 cytokine production [4]. In colonic biopsies from UC patients, CCR3 expression was higher than that in Crohn’s disease (CD) or control biopsies [26]. CC-chemokine ligand 11 (CCL11), often referred to as eotaxin-1, acts as a ligand for CCR3. CCL11 stimulates the migration of eosinophils and Th2 cells through interaction with CCR3 [27]. Moreover, inflammatory diseases, including UC [28], allergic rhinitis [29], AD [30], asthma [31], and IBD [32] express high levels of CCL11. IL-5 promotes eosinophil production through IL-5Rα and contributes to the pathogenesis of IBD, while IL-5 receptor antagonists alleviate DSS colitis [33,34]. Eosinophils also express toll-like receptors (TLRs). TLR-mediated activation of eosinophils is implicated in UC [35].

This study aimed to assess whether TA induces immune responses that exacerbate Th2-dominant diseases. Additionally, it examined the expression of CCR3, CCL11, IL-5Rα, and TLRs in TA-treated normal and colitis mice to evaluate the role of TA in enhancing eosinophil-mediated inflammation through the modulation of these receptors and ligands. This study included the evaluation of dose-dependent effects of TA on eosinophil activation and Th2 cytokine responses in normal and colitis mice, with a focus on its role in immune modulation. Eosinophil activation markers were assessed in the spleen, peripheral blood leukocytes (PBLs), and LNs, alongside Th2 cytokines and IL-17 levels. Additionally, the expressions of eosinophil-regulatory molecules were quantified. This study suggests the need for further exploration of the mechanisms by which TA regulates eosinophil activity and Th2 cytokine production, which may have implications for the management of immune-related diseases such as UC.

## 2. Materials and Methods

### 2.1. Mice

In this study, 44 8-week-old male BALB/c mice were purchased from Orient Bio (Seongnam-si, Gyeonggi-do, Republic of Korea). All mice were housed in a specific pathogen-free (SPF) facility. The environmental conditions were meticulously adjusted with a temperature range of 20–23 °C and a humidity level of 50–60%. Ethical approval for all experimental protocols was strictly reviewed and granted by the Institutional Animal Care and Use Committee (IACUC) of Ajou University (IACUC approval number: AMC-2023-0022). All procedures in animal experiments were performed in strict accordance with relevant regulations and instructions. Before starting the experiment, all mice were acclimated to standard environmental conditions for 7 days. During this acclimation period, mice had unrestricted access to standard food and autoclaved water. All mice were carefully monitored throughout the experimental work.

### 2.2. Experimental Design and Dextran Sodium Sulfate (DSS)-Induced Colitis

Mice were divided into eight groups, with five to six mice in each group. Groups 1 to 4 were experiments to study the effects of various doses of tartaric acid (TA) (Sigma–Aldrich, St. Louis, MO, USA). Group 1 was the normal healthy control group. Group 2 was the low-dose TA group and received 5 µg TA. Group 3 was the medium-dose TA group and was administered 25 µg TA, and group 4 was the high-dose TA group and was administered 50 µg TA. TA was administered orally. Groups 5–8 were experiments to examine the role of TA in the colitis model. Group 5 was a healthy normal control, group 6 was healthy normal mice administered 50 µg TA, group 7 was administered DSS (MP Biomedicals, Irvine, CA, USA) to induce colitis, and group 8 was administered 50 µg TA simultaneously with DSS. DSS was used at a concentration of 4% and administered via drinking water. The administration period for all groups was 10 days. Body weights were recorded daily during this period. On day 11, all mice were sacrificed, and PBL, LN, spleen, and intestine samples were collected and further analyzed.

### 2.3. Preparation of Single-Cell Suspensions

Single-cell suspensions were made from PBLs, spleen, and LNs for flow cytometric analysis. For PBL collection, red blood cell lysis was performed using ammonium-chloride-potassium (ACK) lysis buffer (150 mM NH_4_Cl, 1 mM KHCO_3_, and 0.1 mM EDTA) at 37 °C for 5 min. Cells were then rinsed with autoclaved PBS (pH 7.2). Whole spleen and LN tissues were isolated from individual mice and gently minced with a 70 μm mesh strainer to obtain a fine suspension of individual cells. The spleen tissue was then treated with ACK buffer to lyse red blood cells. To eliminate any remaining lysed cells and ACK buffer, splenocytes and LN cells were rinsed with PBS.

### 2.4. Flow Cytometric Analysis

The flow cytometry procedure was performed according to the protocol previously established [36]. In brief, 1 × 10^6^ cells from PBLs, LNs, and splenocytes were incubated separately with anti-mouse antibodies, such as efluor450-conjugated CD11b (eBioscience, Cat# 48-0112-82 San Diego, CA, USA), PE-conjugated SiglecF (eBioscience, Cat# 12-1702-82), and APC-conjugated CCR3 (BD Biosciences, Cat# 747820) at 4 °C for 30 min in the dark to assess the surface expressions. After staining, cells were rinsed twice with PBS to remove residual unbound antibodies before being resuspended in 300 µL of PBS.

For intracellular staining of PBLs and LNs, 2 × 10^6^ cells were cultured in 12-well plates with RPMI medium supplemented with 1 μg/mL brefeldin A (eBioscience) at 37 °C in a humidified atmosphere containing 5% CO_2_ for 4 h to block cytokine secretion. After incubation, cells were washed with PBS. These cells were further treated with Cytofix/Cytoperm buffer (BD Biosciences, San Diego, CA, USA) for fixation and permeabilization. Afterwards, cells were stained with FITC-conjugated IL-4 (eBioscience, Cat# 11-7042-82), efluor450-conjugated IL-13 (eBioscience, Cat# 48-7133-82), PE-conjugated IL-31(BD Biosciences, Cat# 160704), and PE-cyanine7-conjugated IL-17 (eBioscience, Cat# 25-7177-82) anti-mouse antibodies in Perm/Wash buffer (BD Biosciences) for 40 min at room temperature. After staining, cells were washed with PBS. Surface and intracellular stained cells were examined by Cytek Aurora flow cytometer (Cytek Biosciences, Fremont, CA, USA) for data acquisition. Gating was performed by initially excluding debris from the cell population according to forward-scatter area (FSC-A) versus side-scatter area (SSC-A). Subsequently, the identification of single cells was achieved by comparing forward-scatter height (FSC-H) with FSC-A.

### 2.5. RNA Extraction and Real-Time Quantitative PCR (qRT-PCR)

Total RNA was extracted using TRIzol (Thermo Fisher, Waltham, MA, USA) according to the manufacturer’s instructions using homogenized splenocytes. Subsequently, 1 µg of total RNA was pre-transcribed using the Prime Script cDNA Synthesis kit (Takara Shuzo Co., Otsu, Shiga, Japan) to obtain first-strand cDNA. Each target gene was analyzed in duplicate by qRT-PCR with SYBR Green PCR Master Mix (Applied Biosystems, Foster City, CA, USA) using a 7500 Real-Time PCR System (Applied Biosystems). In qRT-PCR, a 20 µL reaction volume was utilized together with a 1 µL cDNA template. The following were the cycle conditions: an initial denaturation at 94 °C for 2 min, followed by 40 cycles of 94 °C for 3 s, 55 °C for 30 s, and 72 °C for 30 s, with a final extension at 72 °C for 10 min. The 2^−ΔΔCt^ method was used to assess the relative gene expression levels [37]. To determine relative expression ratios, mRNA for each gene was normalized to the housekeeping gene β-actin and compared to the control. The results are expressed as fold changes relative to the controls. The primer sequences used in this experiment are summarized in Table 1.

### 2.6. Transmission Electron Microscopy

LNs were obtained from each mouse and processed for transmission electron microscopy (TEM) using a routine method. The collected LN tissues were first immersed in Karnovsky’s fixative solution for more than 2 h, then washed with PBS and postfixed with 1% osmium tetroxide for 30 min to further stabilize the tissues. The samples were then rinsed with PBS. The tissues were sequentially dehydrated in ethanol. The dehydrated tissues were embedded in Epon-Araldite resin to make a solid block containing the tissues. The resin-embedded tissues were cut into ultrathin sections using an ultramicrotome (Reichert Jung Ultracut S (Leica, Vienna, Austria) and then placed on copper grids. These thin sections were treated with lead citrate and uranyl acetate for staining. These stained sections were examined and analyzed by Zeiss electron microscope (Leo, Oberkohen, Germany) (A = SE1, WD = 2.9 mm, EHT = 28.00 kv).

### 2.7. Statistical Analysis

Statistical comparison between experimental groups was assessed by performing an unpaired Student’s *t*-test. Data are presented as mean ± standard deviation (SD). Results were considered statistically significant if the *p*-value was less than 0.05. All analyses were carried out with GraphPad Prism (version 8.3.1) for Windows (GraphPad Software, La Jolla, CA, USA).

## 3. Results

### 3.1. Expression Levels of Eosinophil Activation Markers in PBLs of Normal Mice Treated with Tartaric Acid (TA)

**Figure 1:**
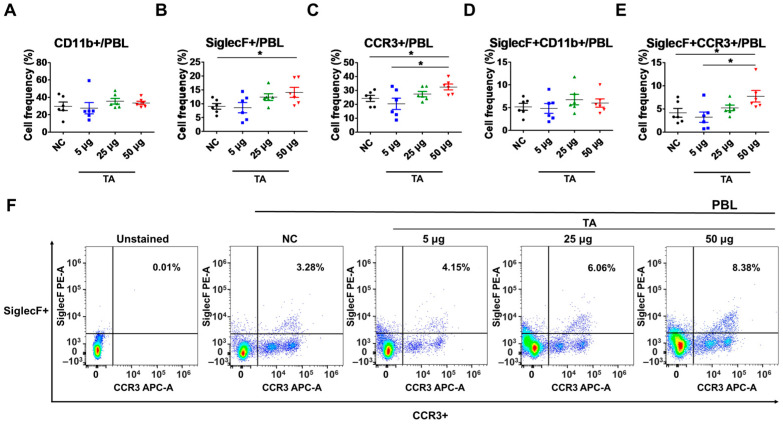
TA administration impacts the expression of SiglecF+, CCR3+, and SiglecF + CCR3+ eosinophil markers in peripheral blood leukocytes of 8-week-old mice (n = 6). Eosinophil markers were evaluated by flow cytometry after TA administration at doses of 5 µg, 25 µg, or 50 µg in normal mice. (**A**–**E**) show the expression levels of eosinophil activation markers in untreated normal control mice and mice administered various doses of TA. (**F**) shows representative flow cytometry plots of SiglecF + CCR3+ eosinophil markers in PBLs. Statistical significance was assessed using Student’s *t*-test. Data are presented as means, and error bars represent standard deviations. * *p* < 0.05.

The frequencies of various eosinophil markers were analyzed in both untreated and TA-treated normal mice. Specifically, the presence of CD11b+, SiglecF+, CCR3+, SiglecF + CD11b+, and SiglecF + CCR3+ cells were assessed in PBLs collected from 8-week-old mice by flow cytometry. Normal mice administered 50 µg TA had significantly higher frequencies of SiglecF+ (*p* < 0.05), CCR3+ (*p* < 0.05), and SiglecF + CCR3+ (*p* < 0.05) compared to those of the normal controls (Figure 1B,C,E). In addition, the frequencies of CCR3+ (*p* < 0.05) and SiglecF + CCR3+ (*p* < 0.05) cells were significantly increased in mice treated with 50 µg TA as opposed to mice administered 5 µg TA (Figure 1C,E). These results indicate that TA is important for inducing eosinophil responses, as indicated by the frequencies of SiglecF+, CCR3+, and SiglecF + CCR3+ cells. The frequencies of CD11b+ and SiglecF + CD11b+ cells, another marker associated with eosinophil activation, did not differ significantly between the control and TA-treated groups (Figure 1A,D). Figure 1F shows the representative flow cytometry plots of SiglecF + CCR3+ cells to identify and quantify the eosinophil populations within the PBLs.

### 3.2. Increased Frequencies of SiglecF+ Cells in LNs of TA-Treated Mice

**Figure 2:**
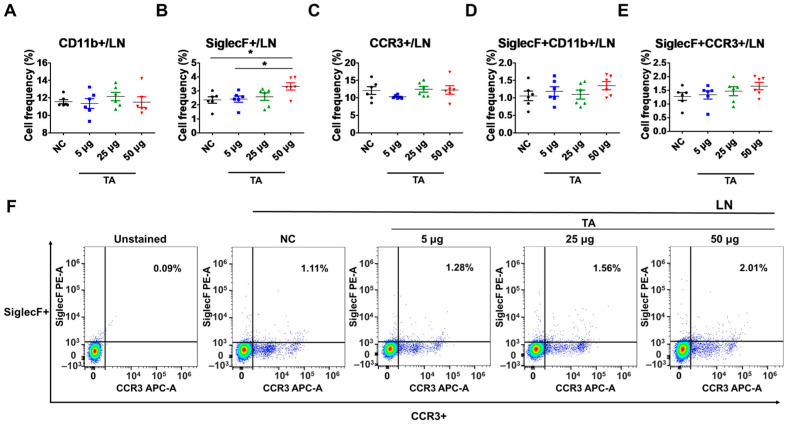
Effects of TA on eosinophil activation markers in lymph nodes of 8-week-old mice (n = 6 per group). Eosinophil markers were evaluated by flow cytometry in LNs after mice were administered TA at doses of 5 µg, 25 µg, or 50 µg. (**A**–**E**) show the relative expression of eosinophil activation markers in the different treatment groups. (**F**) shows representative flow cytometry plots of SiglecF + CCR3+ eosinophil markers in LNs. Statistical significance was assessed using Student’s *t*-test. Data are presented as means, and error bars represent standard deviations. * *p* < 0.05.

To further examine the effects of TA on eosinophil activation within LNs, the frequencies of CD11b+, SiglecF+, CCR3+, SiglecF + CD11b+, and SiglecF + CCR3+ cells were assessed in 8-week-old TA-treated mice using flow cytometry. This study detected a significant upregulation in the frequencies of SiglecF+ cells in mice administered 50 µg TA compared to those in the normal controls (*p* < 0.05) and the mice receiving 5 µg TA (*p* < 0.05), showing the dose-dependent effects of TA on SiglecF+ eosinophils (Figure 2B). This indicates that higher doses of TA selectively enhance SiglecF+ eosinophil expression within LNs, and the proliferation of these SiglecF+ eosinophils is more prominent at higher TA doses. Other markers, such as CD11b+, CCR3+, SiglecF + CD11b+, and SiglecF + CCR3+, did not show significant differences between normal control and TA-treated mice (Figure 2A,C,D–F).

### 3.3. Upregulation of SiglecF+ and SiglecF + CCR3+ Expressions in the Spleens of TA-Treated Mice

**Figure 3:**
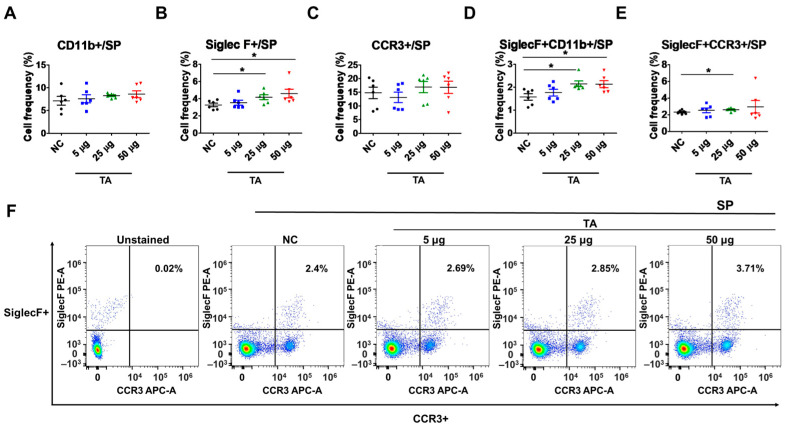
Influence of TA on the expression of SiglecF+ and SiglecF + CCR3+ eosinophil markers in the spleen of 8-week-old mice (n = 6 per group). Eosinophil markers were analyzed by flow cytometry after administering TA to normal mice at doses of 5 µg, 25 µg, or 50 µg. (**A**–**E**) show the frequencies of eosinophil activation marker-positive cells under different treatment conditions. (**F**) shows representative flow cytometry plots of SiglecF + CCR3+ cells in the spleen. Statistical significance was assessed using Student’s *t*-test. Data are presented as means, and error bars represent standard deviations. * *p* < 0.05.

The influence of TA on splenic eosinophils in mice was assessed by measuring the frequencies of CD11b+, SiglecF+, CCR3+, SiglecF + CD11b+, and SiglecF + CCR3+ cells using flow cytometry. The results revealed a significant increase in the frequencies of SiglecF+ cells (*p* < 0.05) and SiglecF + CD11b+ cells (*p* < 0.05) in mice treated with 50 µg of TA compared to the control group (Figure 3B,D). Furthermore, in mice treated with 25 µg of TA, the frequencies of SiglecF+ cells (*p* < 0.05), SiglecF + CD11b+ cells (*p* < 0.05), and SiglecF + CCR3+ cells (*p* < 0.05) were also significantly elevated compared to the those of the control group (Figure 3B,D–F). These findings suggest that medium and high doses of TA markedly enhance splenic eosinophil proliferation and activation. No significant changes were observed in the frequencies of single-positive CD11b+ or CCR3+ cells in any group (Figure 3A,C).

### 3.4. Increased Th2 Cytokine Expression in TA-Treated Mice

**Figure 4:**
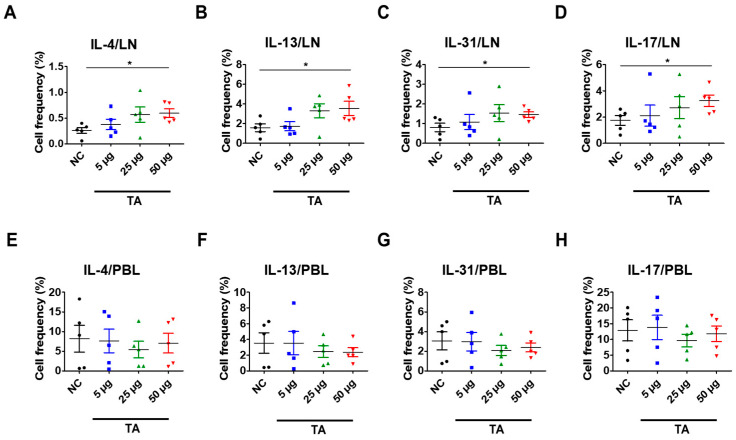
TA influences Th2 cytokine expressions in 8-week-old mice (n = 5 per group). Th2 cytokines (IL-4, IL-13, IL-31) and IL-17 were measured by flow cytometry in LNs and PBLs after TA administration to normal mice at doses of 5 µg, 25 µg, or 50 µg. (**A**–**D**) show higher expressions of IL-4, IL-13, IL-31, and IL-17 in the LNs of mice treated with 50 µg TA compared to mice treated with low and medium TA doses (5 µg and 25 µg) and normal controls. In PBLs, there were no significant differences in the frequencies of cytokine-positive cells (**E**–**H**). Statistical significance was assessed using Student’s *t*-test. Data are presented as means, and error bars represent standard deviations. * *p* < 0.05.

Eosinophils have been extensively linked to Th2 immunity [38]. To further study the Th2 response, which is closely associated with eosinophil activation and is also affected by TA treatment, the frequencies of Th2 cytokine-producing cells in the LNs and PBLs of untreated normal mice and TA-treated mice were analyzed. These cytokines were significantly increased in the LNs of normal mice treated with 50 µg TA compared to the normal control group (Figure 4A–D). IL-4 and IL-13, which are important for Th2 cell maturation and eosinophil recruitment in LNs, were significantly elevated in 50 µg TA-treated mice compared with the normal control group (*p* < 0.05), reflecting an enhanced Th2 response (Figure 4A,B). Additionally, the frequencies of IL-31 expressing cells were also significantly increased in the 50 µg TA group (*p* < 0.05) (Figure 4C), which is consistent with its role in promoting inflammation and pruritus. IL-17, a cytokine known to play a role in inducing Th2 immune responses [39], was significantly increased in this 50 µg TA group (*p* < 0.05) (Figure 4D). This suggests that TA is also involved in IL-17 production and thus in immune regulation. Interestingly, the lower TA doses (5 µg and 25 µg) did not elicit such pronounced cytokine responses, indicating that the dose of TA is important for activating the Th2-mediated immune response. No significant differences were detected in the PBLs from untreated and TA-treated mice (Figure 4E–H).

### 3.5. Changes in IL-5, IL-5Rα, and CCL11 mRNA Expression After TA Administration to Mice

**Figure 5:**
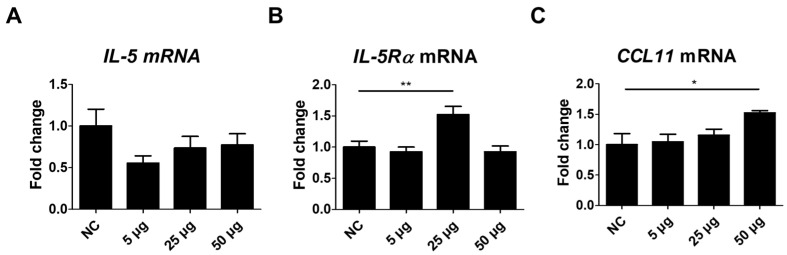
Analysis of IL-5, IL-5Rα, and CCL11 expression in mice administered TA at doses of 5 µg, 25 µg, or 50 µg using real-time qPCR. mRNA expression levels of IL-5, IL-5Rα, and CCL11 were measured via qRT-PCR in 8-week-old mice. (**A**–**C**) show the expression levels of IL-5, IL-5Rα, and CCL11 in untreated and TA-treated mice (n = 6). Statistical significance was assessed using Student’s *t*-test. Data are presented as means, and error bars represent standard deviations. * *p* < 0.05, ** *p* < 0.01.

To further address the effects of TA on eosinophils, the expressions of IL-5, IL-5Rα, and CCL11 were assessed in the spleen tissues of TA-treated mice. IL-5, a pro-inflammatory cytokine involved in the development, activation, and trafficking of eosinophils via the IL-5Rα [40] showed no significant difference between the untreated and TA-treated mice (Figure 5A). However, IL-5Rα expression levels were significantly enhanced in mice treated with 25 µg TA relative to the normal controls (*p* < 0.01) (Figure 5B). No increase in IL-5Rα was observed in mice treated with 50 µg TA. This suggests that 25 µg TA is adequate to increase IL-5Rα in mice, and increasing the dose does not directly correlate with an increase in receptor expression levels.

CCL11, a chemokine that selectively attracts eosinophils to the site of inflammation and contributes to polarized Th2 immune responses [31], was significantly increased in mice administered 50 µg TA (*p* < 0.05) (Figure 5C). Elevated CCL11 levels in the spleen tissues of 50 µg TA-treated mice suggest an enhanced chemotactic environment that may contribute to Th2 immune responses by promoting eosinophil accumulation and activation. The expression of IL-5, IL-5Rα, and CCL11 was unchanged in 5 µg TA-treated mice (Figure 5A–C), indicating that low-dose TA did not affect eosinophil activation. Collectively, these results suggest that IL-5Rα and CCL11 were upregulated in response to TA treatment, emphasizing a possible role for IL-5Rα and CCL11 in mediating eosinophil activation in mice.

### 3.6. Eosinophil Identification in LNs of TA-Treated Mice by Transmission Electron Microscope

**Figure 6:**
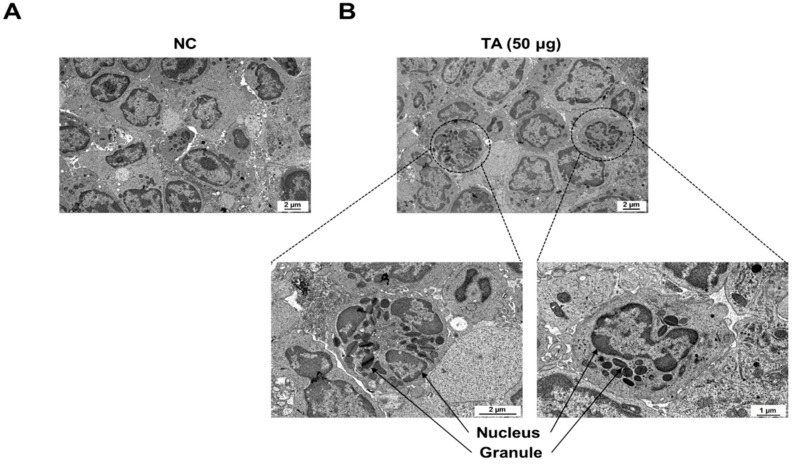
Electron microscopic images of immune cells in lymph nodes of 8-week-old mice. (**A**) LNs from normal control mice without eosinophils. (**B**) LNs from mice administered 50 µg TA showing eosinophils. Images highlight the presence and morphology of eosinophils in the treated group in contrast to the control group.

TEM showed a marked increase in the number of eosinophils in the LNs of mice treated with 50 µg TA (Figure 6B). High-resolution images clearly show typical eosinophils characterized by granular cytoplasm and two-lobed nuclei in the LNs of mice treated with 50 µg TA. The group treated with 50 µg of TA exhibited 6 to 8 eosinophils (average of 7) per 3.05 mm 200-mesh grid. In contrast, no eosinophils were detected in the LNs of the normal control group (Figure 6A). This observation suggests that daily administration of 50 µg TA for 10 days plays a role in eosinophil production in the LNs.

### 3.7. Effects of TA on DSS-Induced Colitis in Mice

**Figure 7:**
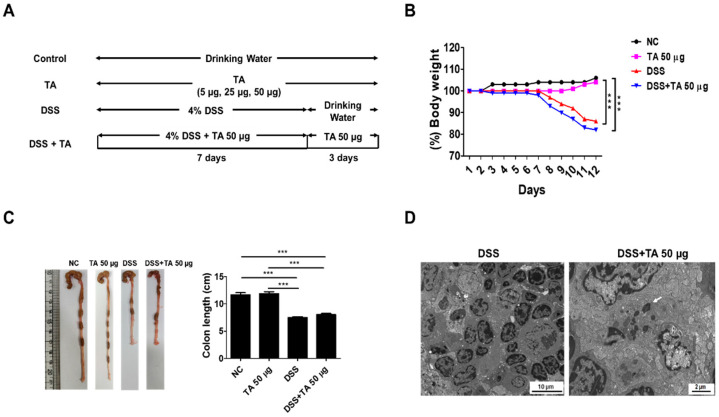
Influence of TA in DSS-induced colitis model. The experiment was performed using 8-week-old mice (n = 5 per group). (**A**) Experimental groups and dosing schedule. (**B**) Trends in body weight changes. (**C**) Comparison of colon lengths. Data are expressed as mean ± SD, and error bars represent standard deviations. (**D**) TEM images of the LNs of colitis mice administered with TA and the LNs of colitis mice not administered with TA. The arrow indicates eosinophil granule. *** *p* < 0.001.

A DSS model was constructed to evaluate the effects of TA in colitis mice (Figure 7A). To induce colitis, 4% DSS was provided in the drinking water for 7 days, followed by regular water for 3 days. To determine the effects of TA, a separate group of mice was given 50 µg TA together with DSS for the first 7 days, and then 50 µg TA alone for the next 3 days. Body weight was tracked throughout the study (Figure 7B). Colitis mice administered TA showed a decrease in body weight, especially compared with the normal control group and the group treated with 50 µg TA. No significant difference in body weight was observed between the DSS group and DSS group administered 50 µg TA. Similarly, colon length was reduced in both colitis groups compared with the that in the control groups, and there was no difference between colitis mice treated with or without TA (Figure 7C). In the LNs of colitis mice administered TA, 1 to 3 (average 2) eosinophils per 3.05 mm 200-mesh grid were observed with early-stage granules by TEM, but no eosinophils were found in the LNs of colitis mice not administered TA (Figure 7D). TA can accelerate the appearance of eosinophils in mice with colitis.

### 3.8. mRNA Expression of Eosinophil-Related Regulatory Molecules After TA Administration to Mice with Colitis

**Figure 8:**
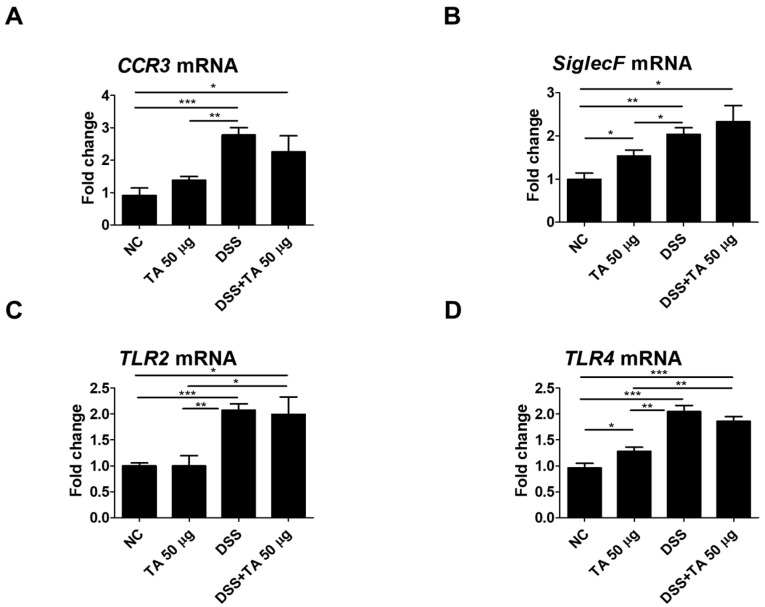
Analysis of mRNA expression of eosinophil-related regulatory molecules in the spleen of colitis mice following TA administration by qRT-PCR (n = 5 per group). (**A**) CCR3, (**B**) SiglecF, (**C**) TLR2, and (**D**) TLR4. Statistical significance was determined using Student’s *t*-test. Bars represent mean expression levels, and error bars represent standard deviation. * *p* < 0.05, ** *p* < 0.01, *** *p* < 0.001.

Several regulatory molecules play a key role in the recruitment of eosinophils into tissues during inflammatory responses [41]. To identify eosinophil-related regulatory molecules in colitis mice according to TA treatment, mRNA levels of CCR3, SiglecF, TLR2, and TLR4 were quantified in the spleen. Untreated colitis mice and 50 µg TA-treated colitis mice exhibited significantly increased expression levels of CCR3 (*p* < 0.001 and *p* < 0.05, respectively) and SiglecF (*p* < 0.01 and *p* < 0.05, respectively) compared to those of the normal control group (Figure 8A,B). TLR2 expression levels were significantly higher in both colitis (*p* < 0.001) and TA-administered colitis (*p* < 0.05) mice compared to the normal control group (Figure 8C). TLR4 expression levels were also increased in both colitis and TA-administered colitis mice compared to the normal control group (*p* < 0.001). In normal mice administered TA, the expressions of SiglecF (*p* < 0.05) and TLR4 (*p* < 0.05) were significantly increased compared to those of normal mice that were not administered TA (Figure 8B,D). TLR2 and TLR4 expressions were significantly higher in colitis mice compared to those in TA-administered normal mice. However, there was no significant difference in CCR3, SiglecF, TLR2, and TLR4 expression depending on whether or not TA was administered in colitis mice. This suggests that 50 µg of TA administration for 10 days did not significantly affect eosinophil-related regulatory molecules in colitis mice.

### 3.9. Th2 Cytokine mRNA Expression by TA Administration in Colitis Mice

**Figure 9:**
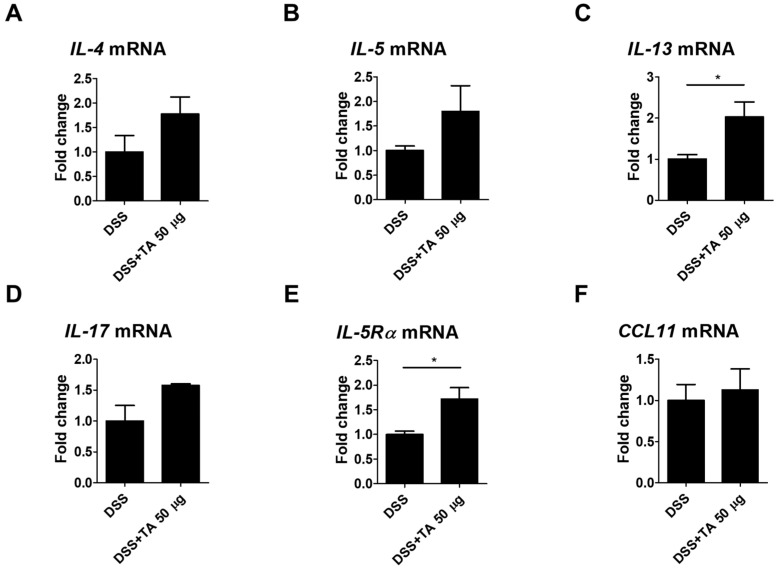
TA administration modulated mRNA expression of eosinophil-associated cytokines in colitis mice (n = 5 per group). qRT-PCR was used to measure mRNA expression levels of genes. (**A**) IL-4, (**B**) IL-5, (**C**) IL-13, (**D**) IL-17, (**E**) IL-5Rα, and (**F**) CCL11. Statistical significance was determined using Student’s *t*-test. Bars represent mean expression levels, and error bars represent standard deviation. * *p* < 0.05.

Eosinophil activation is influenced by Th2 cytokines, and their levels were elevated in the intestinal tissues of patients with UC [42]. The mRNA expression levels of Th2 cytokines as well as IL-17, IL-5Rα, and CCL11 were analyzed in TA-treated and TA-non-treated colitis mice. IL-13 is known to induce inflammation and contribute to UC pathology [43]. IL-13 (*p* = 0.05) and IL-5Rα (*p* < 0.05) were significantly increased in the TA-administered colitis group compared with the unadministered colitis group (Figure 9C,E). This suggests that TA treatment may increase these markers and aggravate colitis. The levels of IL-4, IL-5, IL-17, and CCL11 were increased in the TA-administered colitis group compared with the unadministered colitis mice, but this increase was not statistically significant (Figure 9A,B,D,F). These results suggest that TA can selectively amplify specific Th2 cytokine responses, especially IL-13 and IL-5Rα, in colitis, which may negatively affect colitis symptoms.

## 4. Discussion

Eosinophils exhibit a regulatory role in maintaining immune homeostasis [44]. Eosinophilic diseases are well defined by elevated eosinophil numbers either in specific tissues and/or in the blood (eosinophilia), where eosinophils promote tissue damage and inflammation through receptors, chemokines, cytotoxic granules, and cytokines [22]. Eosinophilia and localized eosinophil accumulation are commonly caused by eosinophilic gastrointestinal disorders, including eosinophilic esophagitis and ulcerative colitis (UC) [45,46] and allergic reactions in asthma [47]. TA is a naturally occurring dietary organic acid found mainly in fruits and beverages such as wine [48]. It is also widely used as a food additive, as an antioxidant, and in pharmaceutical formulations [49]. In pharmaceutical formulations for humans, the acceptable daily intake (ADI) for TA is 30 mg/kg body weight (BW) [50]. For rodents, the ADI is 500 mg sodium tartrate/kg BW at which no adverse effects are observed based on gastrointestinal effects, equivalent to 25 mg TA/day per 20 g mouse [51]. However, the specific effects of TA on eosinophilic and Th2 cytokine responses have not been systematically investigated.

The present work aimed to provide important insights into the effects of TA on eosinophil activation and Th2 cytokine responses in normal healthy and colitis mice, specifically, with a main focus on how different doses of TA have distinct immunological effects in these mice. The current research has found TA exerts a dose-dependent effect on eosinophil activation makers, Th2 cytokines, IL-17, and eosinophil-related regulatory molecules such as TLR2, TLR4, IL-5Rα, and CCL11. The high dose (50 µg) of TA significantly alters the expression levels of eosinophil activation markers, promoting Th2 cytokine profiles and IL-17, TLR4, and CCL11 expression levels in normal, healthy control mice. This broad upregulation suggests that TA primes the immune response even in the absence of inflammation. Moreover, colitis mice treated with 50 µg TA exhibited significant increases in IL-13 and IL-5Rα levels, which likely contribute to the exacerbation of colitis symptoms rather than promoting resolution. Additionally, we directly visualized eosinophils in LNs using TEM, providing structural evidence that high-dose TA treatment promotes eosinophil proliferation and activation. These findings underscore the potential of TA to modulate immune responses in both healthy and inflammatory conditions, offering new insights into its role in eosinophil-driven inflammation.

CCR3 is a G protein-associated membrane receptor largely present on eosinophils and related to UC and allergic diseases [23,24,26]. It is additionally found on Th2 cells and plays a role in their development and the secretion of Th2 cytokines [23]. Researchers using CCR3 knockout mice have found that reduced CCR3 expression inhibited eosinophil infiltration and reduced the release of Th2 cytokines [4]. In the current research, upregulation in the frequencies of CCR3+ cells was noticed in the PBLs of normal mice treated with 50 µg TA compared to those of the controls. CCR3 expression in colonic biopsies from UC patients was higher and more frequent than in the controls, regardless of disease activity [26]. However, in this study, no difference in CCR3 mRNA levels was observed between TA-treated and untreated colitis mice. SiglecF, a glycan-binding protein prominently expressed on mature circulating mouse eosinophils, is known to regulate eosinophilic inflammation [52]. In our study, treatment with 50 µg TA in normal mice led to significantly enhanced frequencies of SiglecF+ cells in LNs compared to those in the normal control group and 5 µg TA-treated group. At this dose, SiglecF+ and SiglecF + CCR3+ cell frequencies were significantly raised in the PBLs and spleens of normal mice. This increase was not detected in normal control mice or in normal mice administered with 5 µg TA. In colitis mice, SiglecF+ expression was not significantly influenced in TA-administered colitis mice compared to untreated colitis mice. Upregulation of these eosinophil markers in normal mice administered TA suggests that higher concentrations of TA promote eosinophil proliferation or generation, thereby increasing recruitment and activation. There are several studies on TA and disease, including studies on its ability to improve experimental nonalcoholic fatty liver disease [15], its ability to improve polycystic ovary syndrome (PCOS) in animal models of PCOS [53], and its potential to exert antihypertensive and vasorelaxant effects in rodents [54]. Regarding the negative effects of tartaric acid, one study showed that inhaled tartaric acid lowered the cough sensitivity threshold in patients with bronchitis [55], but other than that, there are few studies on its negative functions [16]. We have not yet found any reports linking SiglecF and TA.

Eosinophils can amplify Th2-mediated immune responses by secreting various cytokines, such as IL-4, IL-5, IL-13, IL-31, and IL-17. These cytokines play a central role in eosinophil activation and recruitment and are essential for Th2 inflammation [38]. Previous animal studies have shown that reduced IL-4 levels correspond with decreased eosinophilia, emphasizing its role in eosinophil regulation [56]. Additionally, IL-13 is known to promote eosinophil survival, activation, and trafficking, as demonstrated in animal models of eosinophilic inflammation [57]. Imbalances in IL-4 and IL-13 are implicated in the development of inflammatory diseases like UC and asthma [58]. Eosinophils are a significant source of IL-31 and IL-17, which, beyond their roles in allergic inflammation, also contribute to the deterioration of colitis at higher levels by intensifying eosinophil-driven inflammation [42,59,60]. In the current study, treatment of normal mice with 50 µg TA led to increased levels of IL-4, IL-13, IL-31, and IL-17 compared to normal, healthy controls. In colitis mice, however, only IL-13 showed a pronounced increase following 50 µg TA treatment compared to untreated colitis mice, suggesting a selective enhancement of this cytokine in the inflammatory setting. Moreover, TEM data complement the flow cytometry results by providing detailed structural evidence of eosinophil accumulation within the LNs, further emphasizing the role of TA in promoting eosinophil infiltration and contributing to the inflammatory response.

Eosinophils express receptors for multiple chemokines and cytokines, enabling them to play their role in inflammatory responses and maintain homeostasis [61,62,63]. Eotaxin-1/CCL11 is an inflammatory chemokine primarily renowned for its contribution to attracting eosinophils to inflammatory sites [31]. In addition, CCL11 levels are elevated in both the serum and colonic tissues of patients with UC and are also increased in the colitis model [28,64]. CCL11 interacts with the CCR3 receptor present on eosinophils and induces eosinophil migration both in vitro and in vivo [65,66]. Additionally, CCL11 is involved in promoting the Th2 immune response [31]. The findings of the present study showed that CCL11 mRNA levels were significantly increased in the spleens of normal mice treated with 50 µg TA. The increased CCL11 mRNA expression suggests a chemotactic environment created by TA, which may facilitate the selective migration of eosinophils into tissues and further amplify the Th2 response. Although CCL11 expression was also higher in colitis mice treated with 50 µg TA compared to untreated colitis mice, this increase did not reach statistical significance. Additionally, the production and activation of eosinophils are significantly influenced by IL-5 through IL-5Rα [67]. Our study showed that IL-5 levels in normal and colitis mice did not significantly differ depending on whether TA was administered. In IL-5Rα, IL-5Rα mRNA levels were significantly increased in normal mice treated with 25 µg TA compared to 5 µg TA-treated and control mice. Interestingly, upregulation in IL-5Rα expression was observed in colitis mice treated with 50 µg TA compared to untreated colitis mice, suggesting that the effect of TA on eosinophil activation may be dose-dependent, with higher doses likely to correlate with increased receptor expression and thus enhance eosinophil functions.

Moreover, TLRs and TLR-mediated activation of these cells have been implicated in the pathogenesis of colitis [35]. In this study, increased mRNA expressions of TLR2 and TLR4 were observed in the DSS colitis group compared to those in the normal group. In normal mice, TA administration increased the expression of TLR4, while in colitis mice, TA did not affect the mRNA levels of TLR2 and TLR4. This suggests that 50 µg TA is not sufficient to further activate the pathway for TLR expression when inflammation is already established. In addition, TLR4 expression in TA-administered normal mice was significantly higher than that in non-administered normal mice, suggesting that higher doses of TA may be required to activate the immune system by upregulating TLR4, which is involved in pathogen recognition and the initiation of inflammatory responses [68]. These results suggest that TA has the potential to modulate innate immune receptors such as TLR2 and TLR4. Further studies are needed to understand the mechanisms by which TA affects TLR2 and TLR4 activation in both inflammatory and non-inflammatory conditions.

Despite this, this article has some limitations. First, the present study did not investigate the long-term effects of TA exposure. While the short-term impacts on eosinophil activation and cytokine expression were demonstrated, it remains unclear whether these effects persist over time or lead to any long-term alterations in immune functions. Second, the study focused primarily on a few specific markers, chemokines, cytokines, and receptors associated with eosinophil activation. The roles of other chemokines and cytokines that interact with eosinophils were not explored, which might have revealed further complexities of the effect of TA. Thirdly, the exact mechanism by which TA influences eosinophil activation and Th2 cytokine production in inflammatory and non-inflammatory conditions remains unclear. Lastly, the environmental and genetic factors that might influence the response to TA were not controlled in this study. Additional analysis is required to consider all these factors to deeply understand the context in which TA exerts its effects.

Based on the observed effects of TA on eosinophil activity and Th2 cytokine production in non-inflammatory conditions and inflammatory diseases such as UC, TA may exacerbate conditions associated with eosinophilia and Th2-based immune responses in certain other eosinophil-associated diseases. Future studies should comprehensively evaluate the effects of TA and investigate the underlying mechanisms by which it exacerbates conditions such as UC and other eosinophil-associated diseases. Such studies may provide valuable insights into potential therapeutic strategies, particularly how TA interacts with immune pathways and contributes to disease progression. Furthermore, in-depth studies exploring the effects of TA on human populations, particularly individuals with UC or other IBDs, are essential to determine whether the effects observed in animal models are relevant to humans. Understanding how dietary components such as TA affect gut immune responses could advance our knowledge of disease pathogenesis and aid in the management of UC and related disorders. By pinpointing specific dietary factors that modulate inflammation, these studies have the potential to inform novel nonpharmacological interventions to manage gut inflammation and improve patient outcomes.

## 5. Conclusions

This study demonstrates that TA exerts a dose-dependent effect on eosinophil activation and Th2 cytokine responses in both normal and colitis mice. Specifically, TA administration in normal mice significantly enhanced eosinophil activation markers such as SiglecF+, CCR3+, Th2 cytokines (IL-4, IL-13, and IL-31), IL-17, and eosinophil-related regulatory molecules such as CCL11 and TLR4. In addition, IL-5Rα expression was increased in TA-treated normal mice. These findings suggest that TA induces eosinophil activation and promotes a Th2-driven immune response even in the absence of inflammation. In colitis mice, TA administration increased IL-13 and IL-5Rα levels compared to those in untreated colitis mice, suggesting that TA may exacerbate colitis symptoms. These results emphasize that TA leads to more pronounced eosinophil activity and Th2 cytokine profiles in inflammatory and non-inflammatory conditions. However, further studies are required to elucidate the long-term effects, underlying mechanisms, and precise role of TA in colitis progression, as well as its interactions with immune cells such as macrophages, neutrophils, and regulatory T cells, to better understand its broader implications, particularly in the human population. These findings provide new insights into the potential of TA to influence immune responses and its implications in inflammatory and eosinophil-related diseases, as well as its potential dietary impact on immune regulation.

## Figures and Tables

**Table 1:** Primer sequences for PCR.

Gene	Forward	Reverse
IL-4	GGTCTCAACCCCCAGCTAGT	GCCGATGATCTCTCTCAAGTGAT
IL-5	AGGCTTCCTGTCCCTACTCAT	ATTTCCACAGTACCCCCACG
IL-13	CCTGGCTCTTGCTTGCCTT	GGTCTTGTGTGATGTTGCTCA
IL-17	CCTCACACGAGGCACAAGTG	CTCTCCCTGGACTCATGTTTGC
CCR3	TGATGTTTACTACCTGACTGGTG	TGCCATTCTACTTGTCTCTGGT
CCL11	GAATCACCAACAACAGATGCAC	ATCCTGGACCCACTTCTTCTT
SiglecF	CTCCACAGAAGATGACCATCAGG	CTGTCAGCCATACAGACCAGGC
IL-5Rα	AGAACACTGTGTAGCCCTGTT	ACCTGTCCAGTGAGCTTCTTC
TLR2	CACTGGGGGTAACATCGCTT	GAGAGAAGTCAGCCCAGCAA
TLR4	CGAGAGCCCATGGAACACAT	CCCCTGGAAAGGAAGGTGTC
β-actin	TGTCCACCTTCCAGCAGATGT	AGCTCAGTAACAGTCCGCCTAG

## Data Availability

Data are contained within the article.

## Abbreviations

The following abbreviations are used in this manuscript:

CCL11	CC-chemokine ligand 11
CCR3	CC-chemokine receptor 3
DSS	Dextran sulfate sodium
IL	Interleukin
IL-5Rα	Interleukin-5 receptor alpha
LNs	Lymph nodes
PBLs	Peripheral blood leukocytes
TA	Tartaric acid
TLRs	Toll-like receptors
Th2	Type 2 T cell
UC	Ulcerative colitis
